# An Artificial Intelligence Approach for Test‐Free Identification of Sarcopenia

**DOI:** 10.1002/jcsm.13627

**Published:** 2024-11-08

**Authors:** Liangyu Yin, Jinghong Zhao

**Affiliations:** ^1^ Department of Nephrology, Chongqing Key Laboratory of Prevention and Treatment of Kidney Disease, Chongqing Clinical Research Center of Kidney and Urology Diseases, Xinqiao Hospital Army Medical University (Third Military Medical University) Chongqing China

**Keywords:** artificial intelligence, functional capacity, machine learning, sarcopenia

## Abstract

**Background:**

The diagnosis of sarcopenia relies extensively on human and equipment resources and requires individuals to personally visit medical institutions. The objective of this study was to develop a test‐free, self‐assessable approach to identify sarcopenia by utilizing artificial intelligence techniques and representative real‐world data.

**Methods:**

This multicentre study enrolled 11 661 middle‐aged and older adults from a national survey initialized in 2011. Follow‐up data from the baseline cohort collected in 2013 (*n* = 9403) and 2015 (*n* = 10 356) were used for validation. Sarcopenia was retrospectively diagnosed using the Asian Working Group for Sarcopenia 2019 framework. Baseline age, sex, height, weight and 20 functional capacity (FC)–related binary indices (activities of daily living = 6, instrumental activities of daily living = 5 and other FC indices = 9) were considered as predictors. Multiple machine learning (ML) models were trained and cross‐validated using 70% of the baseline data to predict sarcopenia. The remaining 30% of the baseline data, along with two follow‐up datasets (*n* = 9403 and *n* = 10 356, respectively), were used to assess model performance.

**Results:**

The study included 5634 men and 6027 women (median age = 57.0 years). Sarcopenia was identified in 1288 (11.0%) individuals. Among the 20 FC indices, the running/jogging 1 km item showed the highest predictive value for sarcopenia (AUC [95%CI] = 0.633 [0.620–0.647]). From the various ML models assessed, a 24‐variable gradient boosting classifier (GBC) model was selected. This GBC model demonstrated favourable performance in predicting sarcopenia in the holdout data (AUC [95%CI] = 0.831 [0.808–0.853], accuracy = 0.889, recall = 0.441, precision = 0.475, F1 score = 0.458, Kappa = 0.396 and Matthews correlation coefficient = 0.396). Further model validation on the temporal scale using two longitudinal datasets also demonstrated good performance (AUC [95%CI]: 0.833 [0.818–0.848] and 0.852 [0.840–0.865], respectively). The model's built‐in feature importance ranking and the SHapley Additive exPlanations method revealed that lifting 5 kg and running/jogging 1 km were relatively important variables among the 20 FC items contributing to the model's predictive capacity, respectively. The calibration curve of the model indicated good agreement between predictions and actual observations (Hosmer and Lemeshow *p* = 0.501, 0.451 and 0.374 for the three test sets, respectively), and decision curve analysis supported its clinical usefulness. The model was implemented as an online web application and exported as a deployable binary file, allowing for flexible, individualized risk assessment.

**Conclusions:**

We developed an artificial intelligence model that can assist in the identification of sarcopenia, particularly in settings lacking the necessary resources for a comprehensive diagnosis. These findings offer potential for improving decision‐making and facilitating the development of novel management strategies of sarcopenia.

## 1 Introduction

Sarcopenia is a clinical condition characterized by the age‐related loss of skeletal muscle mass and strength/function [1, 2, 3]. The reported prevalence of sarcopenia has ranged from 5.5% to 25.7%, with a higher incidence among men (5.1%–21.0% in men vs. 4.1%–16.3% in women) [2]. However, the true burden of sarcopenia remains largely underestimated because of limited clinical awareness [4]. It is predicted that the global prevalence of sarcopenia will increase from 50 million in 2010 to 200 million by 2050 [5, 6]. This increasing prevalence represents a significant and urgent global public health concern that must be addressed [5, 6, 7].

Research on sarcopenia has burgeoned in the past 20 years, likely driven by several factors: Firstly, sarcopenia can occur independently [8] and can also develop concurrently in other disease groups [9, 10], thus prompting interdisciplinary attention. Secondly, it has grave physiological and clinical consequences such as disability, falls, cardiovascular events, impaired quality of life and increased mortality [1, 2, 3, 9, 10], whereas effective treatment options remain limited [2]. Thirdly, the assignment of a specific code for sarcopenia (M62.84) in the International Classification of Diseases, Tenth Revision, Clinical Modification (ICD‐10‐CM) signifies a milestone in recognizing sarcopenia as a distinct disease entity [11]. Finally, as the aging population continues to grow, issues related to functional disability will become increasingly prominent [12]. China has the world's largest elderly population, which is estimated to reach 479 million people aged 60 and above by 2050 [13]. It is foreseeable that there will be a continued emergence of clinical recognition and research on the screening, diagnosis, treatment and surveillance of sarcopenia both in China and globally [14].

Because of increasing research interest, at least six diagnostic frameworks for sarcopenia have been introduced in the past decade [2, 3, 15, 16, 17, 18], among which two tools have gained widespread acceptance in both research and clinical settings: the updated guidelines from the European Working Group on Sarcopenia in Older People (EWGSOP2) for Western populations [3] and the 2019 consensus update of the Asian Working Group for Sarcopenia (AWGS) for Asians [2]. These frameworks share similar practical definitions and parameter thresholds of sarcopenia, encompassing assessments of muscle strength, physical performance, and muscle mass components. Notably, the comprehensive diagnosis of sarcopenia necessitates specific requirements [2, 3], including (1) in‐person visits to medical institutions by individuals; (2) handgrip strength (HGS) measurement following standardized protocols [19]; (3) physical performance tests (e.g., 6‐m walking speed, five‐time chair stand test or Short Physical Performance Battery) conducted under the supervision of medical professionals; and (4) measurement of skeletal muscle mass using equipment such as dual‐energy X‐ray absorptiometry (DEXA) or bioelectrical impedance analysis (BIA). This poses challenges for smaller institutions and community/home settings lacking the necessary resources to trigger further assessment, necessary interventions and/or operational surveillance algorithms.

Activities of daily living (ADL) and instrumental activities of daily living (IADL) are widely used scales comprising binary questions assessing an individual's functional capacity (FC) across multiple dimensions [20, 21]. Recent international consensus on sarcopenia highlights that sarcopenia can impair ADLs, IADLs, and other FC metrics [15]. Hence, we hypothesized that these metrics may serve as indicators of the onset of sarcopenia. In this study, we aimed to develop an artificial intelligence approach for identifying sarcopenia primarily based on these FC indicators. Our goal was to create a test‐free, rapid, self‐assessable and online‐deployable decision system, facilitating the development of novel screening and surveillance strategies for sarcopenia.

## 2 Methods

### 2.1 Study Design and Population

This was an observational study using both cross‐sectional and longitudinal data from participants enrolled in the China Health and Retirement Longitudinal Study (CHARLS), an ongoing nationally representative longitudinal survey in China [22]. The study design of the CHARLS has been previously described in detail [23]. In 2011, the CHARLS study recruited participants from 10 257 households located in 150 counties or districts and 450 villages across 28 provinces in China. Through structured questionnaires and in‐person interviews, high‐quality data were collected from a nationally representative sample of Chinese adults aged 45 years and older, covering sociodemographic, lifestyle and health‐related information. Follow‐up surveys were conducted every 2 years after the baseline survey, with collected data weighted to ensure an accurate representation of the national population.

In this study, we conducted a retrospective analysis of the CHARLS survey data from 2011 (baseline), 2013 (follow‐up wave 1) and 2015 (follow‐up wave 2). Inclusion criteria encompassed individuals with available data on age, sex, body height, body weight, ADLs, IADLs other ADL/IADL‐like FC indicators and variables used for diagnosing sarcopenia. Exclusion criteria involved missing data on study variables, consistently applied across all survey waves. A flowchart illustrating subject inclusion is provided in Figure S1. The research protocol received approval from the Ethical Review Committee of Peking University (approval number: IRB00001052‐11015), and all participants provided informed consent. The procedures involving human participants adhered to the principles outlined in the 1964 Helsinki Declaration and its subsequent amendments, or equivalent ethical standards, as well as the ethical standards set by the institutional and/or national research committee. This study followed the Strengthening the Reporting of Observational Studies in Epidemiology (STROBE) guidelines [24].

### 2.2 Data Handling

Age (years), sex, body height, body weight and FC items were extracted from the CHARLS database. Body weight and height were measured using a stadiometer and a digital floor scale, respectively, to the nearest 0.1 cm and 0.1 kg. Body mass index (BMI) was calculated as the weight in kilograms divided by the height in metres squared. BMI (kg/m^2^) was as also categorized as underweight (< 18.5), normal (18.5 to < 24), overweight (24 to < 28) or obese (≥ 28) according to Chinese recommendations [25]. A total of 20 FC items were included for analysis based on the variable availability, including six ADLs (dressing, bathing, eating, bed, toilet and urination), five IADLs (money, medication, shopping, meal and housework) and nine other FC items (jogging 1 km, walking 1 km, walking 100 m, chair, climbing, stooping, lifting 5 kg, picking and arm). Respondents who reported any difficulty in completing these tasks were recorded as positive. Detailed descriptions of the 20 FC items are provided in Table S1.

### 2.3 Diagnosis of Sarcopenia

Sarcopenia was retrospectively defined based on the AWGS 2019 framework [2]. Sarcopenia is diagnosed as low appendicular skeletal muscle mass (ASM) plus low muscle strength and/or reduced physical performance. HGS (kg) was measured in both the dominant hand and nondominant hand using a dynamometer (Model: YuejianTM WL‐1000, Nantong Yuejian Physical Measurement Instrument Co., Ltd., Nantong, China) [23]. Individuals were instructed to squeeze the dynamometer using their maximum strength for two times, and the higher value was recorded. Cut‐offs for indicating low HGS were < 28 kg for men and < 18 kg for women [2]. ASM (kg) was calculated retrospectively using an anthropometric equation previously validated for use in Chinese populations, which demonstrated high agreement with DEXA [26]. ASM was adjusted for height in metres squared to obtain the appendicular skeletal muscle mass index (ASMI, kg/m^2^). Cut‐offs for ASMI were men < 7.0 kg/m^2^ or women < 5.4 kg/m^2^. The five‐time chair stand test was conducted to assess physical performance, with a completion time of ≥12 s indicating low physical performance [2].

### 2.4 Machine Learning (ML) Models

**FIGURE 1:**
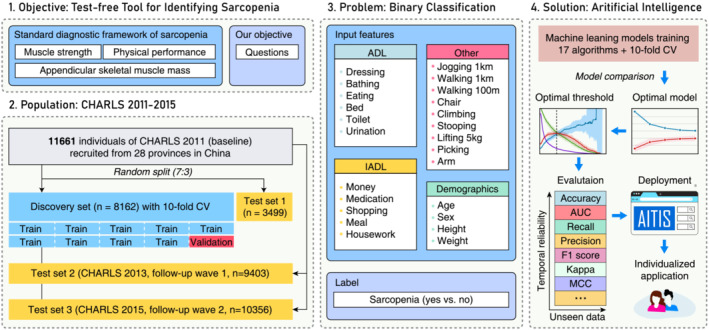
A graphical abstract of the study workflow. CHARLS, the China Health and Retirement Longitudinal Study; CV, cross‐validation; ADL, activities of daily living; IADL, instrumental activities of daily living AUC, area under the curve; MCC, the Matthews correlation coefficient; AITIS, artificial intelligence to identify sarcopenia.

The graphical abstract used to describe the study workflow is shown in Figure 1. Our objective was to create a tool for identifying sarcopenia that is test‐free, rapid, self‐assessable and online‐deployable. The baseline dataset of the CHARLS 2011, consisting of 11 661 individuals with complete data, was shuffled and randomly split into a discovery set (*n* = 8162, 70%) for model training and cross‐validation and test set 1 (*n* = 3499, 30%) for model evaluation. As all input and outcome features were updated in subsequent surveys, individuals with complete study variables in the CHARLS 2013 (*n* = 9403) and 2015 (*n* = 10 356) were used as test sets 2 and 3, respectively. This approach allowed us to simultaneously evaluate the model's generalization performance on new data (test set 1) and its consistency over time (test sets 2 and 3).

The selection of input features was guided by our study hypothesis, primarily including the 20 FC‐related items. We also included age, sex, height and weight, because the four features are all that need to estimate ASM [26], which is the fundamental criterion for diagnosing sarcopenia. Additionally, self‐reported height and weight are reliable [27], thereby supporting self‐assessment. The diagnosis of sarcopenia (yes vs. no) was defined as the binary response variable. Severity of sarcopenia was not considered, as our goal was early identification to prompt further assessment rather than detailed diagnosis.

One‐hot encoding was used to convert categorical data into machine‐recognizable formats [28]. Continuous data were standardized using a *z*‐score approach. Medians and standard deviations from the discovery set were used for standardization in the test sets to prevent information leakage. We trained 17 different ML models independently to predict sarcopenia, chosen for their accessibility and previous usage in similar studies, including our own [29, 30]. Detailed descriptions of the 17 ML algorithms are shown in Table S2. Model performance was assessed using metrics such as accuracy, area under the curve (AUC), recall, precision, F1 score, Kappa and Matthews correlation coefficient (MCC), with higher values indicating better performance. Additionally, sensitivity, specificity, positive predictive value (PPV) and negative predictive value (NPV) were also utilized, as these metrics are more commonly used in clinical context [28, 29].

Ten‐fold cross‐validation with 10 iterations was employed to aggregate model performance metrics, enhancing robustness for comparison and hyperparameter selection. Subsequently, we subjected the optimal ML model to further threshold optimization and feature selection to seek potential performance improvement. Feature selection employed a recursive feature elimination with a 10‐fold cross‐validation (RFECV) approach by setting a weighted F1 score as the evaluation metric. Because the variables of the model are easy to obtain, we have adopted a strategy prioritizing model performance. That is, if the model performance significantly decreases after feature selection, we tend not to reduce the number of features to maintain model performance. The selected model was evaluated in the holdout test set and various subgroups based on age (< 60 vs. ≥ 60) and sex (men vs. women) to assess potential effect modifications.

Feature importance was assessed using the model's built‐in ranking function, whereas model explainability was assessed using a SHapley Additive exPlanations (SHAP) method at both group and individual levels. The final model, named AITIS (Artificial Intelligence to Identify Sarcopenia), was deployed as a web‐based application for prediction and was serialized as a binary file for flexible deployment. Project code and files have been stored online in a GitHub repository (https://github.com/kevinlyy/sarco) for public access.

### 2.5 Statistical Analysis

Continuous data are presented as medians [25th percentile, 75th percentile] and were compared using the nonparametric Wilcoxon's rank‐sum test. Categorical data were expressed as numbers (percentages) and compared using the Chi‐squared test. Differences between correlated (receiver operating characteristic) ROC curves were assessed using Delong's test. Integrated discrimination improvement (IDI) and net reclassification improvement (NRI) were used to assess the discriminatory performance of different predictors. A model‐predicted probability greater than 0.5 was initially defined as the cut‐off point for belonging to the sarcopenia group. Following threshold optimization, probabilities greater than the optimized threshold indicated sarcopenia. All reported *p*‐values were two‐sided and considered significant at *p* < 0.05. All statistical analyses were performed using R (version 4.3.1, Foundation for Statistical Computing, Vienna, Austria) or Python (version 3.9.11, The Python Software Foundation, USA).

## 3 Results

### 3.1 Population Overview

The characteristics of the baseline study population are presented in the overall column of Table 1. There were 11 661 individuals with a median age of 57.0 years, including 5634 men and 6027 women. Among them, 757 (6.5%), 6224 (53.4%), 3403 (29.2%) and 1277 (11.0%) individuals were classified into the underweight, normal weight, overweight and obese groups, respectively. The prevalence rates of low HGS, low ASMI and low physical performance (as indicated by the five‐time chair stand test) were 1950 (16.7%), 2411 (20.7%) and 3182 (27.3%), respectively.

**TABLE 1:** Baseline characteristics of the study population by sarcopenia.

		Sarcopenia		
Characteristics	Overall (*n* = 11 661)	No (*n* = 10 373)	Yes (*n* = 1288)	*P*
Age, years	57.0 [50.0, 64.0]^a^	56.0 [49.0, 62.0]	66.0 [59.0, 74.0]	< 0.001
Sex, men	5634 (48.3)^b^	5051 (48.7)	583 (45.3)	0.022
BMI, kg/m^b^	23.1 [20.8, 25.7]	23.3 [21.2, 25.9]	20.5 [18.8, 23.6]	< 0.001
BMI category				< 0.001
I underweight	757 (6.5)	491 (4.7)	266 (20.7)	
II normal	6224 (53.4)	5494 (53.0)	730 (56.7)	
III overweight	3403 (29.2)	3198 (30.8)	205 (15.9)	
IV obese	1277 (11.0)	1190 (11.5)	87 (6.8)	
Height, m	1.6 [1.5, 1.6]	1.6 [1.5, 1.6]	1.5 [1.5, 1.6]	< 0.001
Weight, kg	58.0 [51.2, 65.8]	58.8 [52.4, 66.5]	49.5 [43.7, 57.5]	< 0.001
Handgrip strength, right, kg	31.4 [25.0, 40.0]	33.0 [27.0, 40.0]	19.0 [15.0, 25.0]	< 0.001
Handgrip strength, left, kg	30.0 [24.0, 38.0]	31.0 [25.1, 39.0]	17.0 [14.0, 24.0]	< 0.001
Handgrip strength, low	1950 (16.7)	662 (6.4)	1288 (100.0)	< 0.001
ASMI, kg/m^b^	6.9 [6.0, 7.6]	6.9 [6.0, 7.6]	6.3 [5.2, 6.9]	< 0.001
ASMI, low	2411 (20.7)	1650 (15.9)	761 (59.1)	< 0.001
Five‐time chair stand test, s	9.9 [8.0, 12.3]	9.6 [7.8, 11.6]	13.5 [11.2, 16.3]	< 0.001
Five‐time chair stand test, abnormal	3182 (27.3)	2277 (22.0)	905 (70.3)	< 0.001
ADL				
Dressing	321 (2.8)	208 (2.0)	113 (8.8)	< 0.001
Bathing	360 (3.1)	239 (2.3)	121 (9.4)	< 0.001
Eating	135 (1.2)	73 (0.7)	62 (4.8)	< 0.001
Bed	312 (2.7)	231 (2.2)	81 (6.3)	< 0.001
Toilet	949 (8.1)	724 (7.0)	225 (17.5)	< 0.001
Urination	335 (2.9)	258 (2.5)	77 (6.0)	< 0.001
IADL				
Money	1153 (9.9)	888 (8.6)	265 (20.6)	< 0.001
Medication	570 (4.9)	437 (4.2)	133 (10.3)	< 0.001
Shopping	622 (5.3)	438 (4.2)	184 (14.3)	< 0.001
Meal	543 (4.7)	382 (3.7)	161 (12.5)	< 0.001
Housework	601 (5.2)	420 (4.0)	181 (14.1)	< 0.001
Other function capacity items				
Jogging 1 km	5542 (47.5)	4624 (44.6)	918 (71.3)	< 0.001
Walking 1 km	944 (8.1)	693 (6.7)	251 (19.5)	< 0.001
Walking 100 m	158 (1.4)	102 (1.0)	56 (4.3)	< 0.001
Chair	2683 (23.0)	2202 (21.2)	481 (37.3)	< 0.001
Climbing	4185 (35.9)	3482 (33.6)	703 (54.6)	< 0.001
Stooping	2936 (25.2)	2429 (23.4)	507 (39.4)	< 0.001
Lifting 5 kg	852 (7.3)	582 (5.6)	270 (21.0)	< 0.001
Picking	264 (2.3)	189 (1.8)	75 (5.8)	< 0.001
Arm	881 (7.6)	680 (6.6)	201 (15.6)	< 0.001

Abbreviations: ADL, activities of daily living; ASMI, appendicular skeletal muscle mass index; BMI, body mass index; IADL, instrumental activities of daily living.

^a^ Median [interquartile range], all such values.

^b^ Number (percentage), all such values.

Functional limitation was most frequently reported for the jogging 1 km (*n* = 5542, 47.5%), climbing (*n* = 4185, 35.9%), stooping (*n* = 2936, 25.2%), chair (*n* = 2683, 23.0%) and money (*n* = 1153, 9.9%) items. In contrast, relatively few difficulties were reported for the eating (*n* = 135, 1.2%), walking 100 m (*n* = 158, 1.4%), picking (*n* = 246, 2.3%), bed (*n* = 312, 2.7%) and dressing (*n* = 321, 2.8%) items.

### 3.2 Sarcopenia

Sarcopenia was found in 1288 (11.0%) individuals. Sarcopenia‐stratified characteristics of the baseline study population are detailed in Table 1. Sarcopenia was significantly associated with higher values/rates of age, low HGS, low ASMI, abnormal five‐time chair stand test results and difficulties across all FC items. Conversely, sarcopenia was associated with lower values/rates of male sex, BMI, body height, body weight, left HGS, right HGS, ASMI and performance on the five‐time chair stand test. Additionally, there were differences in BMI categories between the sarcopenia and nonsarcopenia groups (all *p* < 0.05).

### 3.3 FC for Predicting Sarcopenia

The ability of the 20 FC items to predict sarcopenia was evaluated and compared. The jogging 1 km item exhibited the highest apparent predictive value (AUC = 0.633, 95%CI = 0.620–0.647, sensitivity = 0.713, specificity = 0.554, PPV = 0.166, NPV = 0.940). Statistical tests using Delong's method showed that the jogging 1 km had greater predictive value compared with other 19 items (all *p* < 0.05). IDI analysis showed that jogging 1 km outperformed the other 18 items (all *p* < 0.05) but was inferior to lifting 5 kg (AUC = 0.577, 95%CI = 0.565–0.588, *p* = 0.023). NRI analysis revealed consistent results with Delong's tests, confirming jogging 1 km as optimal among all 20 FC items for predicting sarcopenia (Table 2).

**TABLE 2:** Independent and comparison analyses on the ability of function markers to predict the existence of sarcopenia.

	Diagnostic performance metrics					Intermodel comparison				
Model	AUC (95%CI)	Sensitivity	Specificity	PPV	NPV	*P*‐AUC	IDI (95%CI)	*P*‐IDI	NRI (95%CI)	*P*‐NRI
ADL										
Dressing	0.534 (0.526–0.542)	0.088	0.980	0.352	0.896	< 0.001	0.011 [0.007–0.016]	< 0.001	0.403 [0.347–0.459]	< 0.001
Bathing	0.535 (0.527–0.544)	0.094	0.977	0.336	0.897	< 0.001	0.012 [0.007–0.016]	< 0.001	0.399 [0.342–0.455]	< 0.001
Eating	0.521 (0.515–0.526)	0.048	0.993	0.459	0.894	< 0.001	0.014 [0.009–0.018]	< 0.001	0.465 [0.410–0.519]	< 0.001
Bed	0.520 (0.514–0.527)	0.063	0.978	0.260	0.894	< 0.001	0.022 [0.019–0.025]	< 0.001	0.455 [0.400–0.511]	< 0.001
Toilet	0.552 (0.542–0.563)	0.175	0.930	0.237	0.901	< 0.001	0.014 [0.010–0.017]	< 0.001	0.350 [0.292–0.407]	< 0.001
Urination	0.517 (0.511–0.524)	0.060	0.975	0.230	0.893	< 0.001	0.024 [0.021–0.027]	< 0.001	0.473 [0.418–0.528]	< 0.001
IADL										
Money	0.560 (0.549–0.571)	0.206	0.914	0.230	0.903	< 0.001	0.012 [0.008–0.016]	< 0.001	0.306 [0.248–0.363]	< 0.001
Medication	0.531 (0.522–0.539)	0.103	0.958	0.233	0.896	< 0.001	0.020 [0.017–0.024]	< 0.001	0.412 [0.356–0.468]	< 0.001
Shopping	0.550 (0.541–0.560)	0.143	0.958	0.296	0.900	< 0.001	0.008 [0.004–0.013]	< 0.001	0.347 [0.290–0.404]	< 0.001
Meal	0.544 (0.535–0.553)	0.125	0.963	0.297	0.899	< 0.001	0.011 [0.007–0.015]	< 0.001	0.367 [0.310–0.424]	< 0.001
Housework	0.550 (0.549–0.560)	0.141	0.960	0.301	0.900	< 0.001	0.008 [0.004–0.012]	< 0.001	0.348 [0.291–0.405]	< 0.001
Other items										
Jogging 1 km	0.633 (0.620–0.647)	0.713	0.554	0.166	0.940	Ref	Ref	Ref	Ref	Ref
Walking 1 km	0.564 (0.553–0.575)	0.195	0.933	0.266	0.903	< 0.001	0.006 [0.003–0.010]	0.001	0.281 [0.223–0.338]	< 0.001
Walking 100 m	0.517 (0.511–0.522)	0.043	0.990	0.354	0.893	< 0.001	0.020 [0.016–0.023]	< 0.001	0.467 [0.412–0.521]	< 0.001
Chair	0.581 (0.567–0.594)	0.373	0.788	0.179	0.910	< 0.001	0.014 [0.010–0.017]	< 0.001	0.203 [0.147–0.259]	< 0.001
Climbing	0.605 (0.591–0.619)	0.546	0.664	0.168	0.922	< 0.001	0.009 [0.006–0.012]	< 0.001	0.071 [0.022–0.120]	0.005
Stooping	0.580 (0.566–0.594)	0.394	0.766	0.173	0.910	< 0.001	0.015 [0.012–0.018]	< 0.001	0.197 [0.141–0.253]	< 0.001
Lifting 5 kg	0.577 (0.565–0.588)	0.210	0.944	0.317	0.906	< 0.001	−0.006 [−0.011 to −8 × 10^−4^]	0.023	0.255 [0.197–0.313]	< 0.001
Picking	0.520 (0.513–0.527)	0.058	0.982	0.284	0.894	< 0.001	0.021 [0.018–0.024]	< 0.001	0.454 [0.398–0.509]	< 0.001
Arm	0.545 (0.535–0.555)	0.156	0.934	0.228	0.899	< 0.001	0.017 [0.013–0.020]	< 0.001	0.349 [0.291–0.406]	< 0.001

Abbreviations: ADL, activities of daily living; AUC, area under the curve; CI, confidence interval; IADL, instrumental activities of daily living; IDI, integrated discrimination improvement; NPV, negative predictive value; NRI, net reclassification improvement; PPV, positive predictive value.

### 3.4 Preliminary Model Training, Comparison and Assessment

The distributions of study variables in the discovery and three test sets are presented in Table S3. The rates of positive class (sarcopenia) in the discovery, test set 1, test set 2 and test set 3 were 10.6% (372/3499), 11.2% (916/8162), 11.6% (1091/9403) and 14.5% (1504/10356), respectively. Initially, 17 ML models were trained in the discovery set, with 10‐fold cross‐validated results detailed in Table 3. The gradient boosting classifier (GBC) model exhibited the highest performance, with accuracy = 0.895, AUC = 0.822, recall = 0.224, precision = 0.590, F1 score = 0.321, Kappa = 0.276 and MCC = 0.316. Assessment of the GBC model in the holdout data (test set 1) revealed that the model's performance sustained, with accuracy = 0.901, AUC = 0.831, recall = 0.212, precision = 0.590, F1 score = 0.312, Kappa = 0.271 and MCC = 0.313. Details of the hyperparameters for the GBC model are provided in Table S4.

**TABLE 3:** Model development and validation.

Model	Accuracy	AUC	Recall	Precision	F1	Kappa	MCC
Discovery set (no./events = 8162/916)^a^							
Gradient Boosting Classifier	0.895	0.822	0.224	0.590	0.321	0.276	0.316
Ada Boost Classifier	0.894	0.813	0.222	0.581	0.318	0.272	0.312
Logistic Regression	0.894	0.810	0.165	0.614	0.257	0.220	0.277
Linear Discriminant Analysis	0.889	0.807	0.213	0.519	0.299	0.250	0.281
Light Gradient Boosting Machine	0.892	0.803	0.237	0.542	0.328	0.279	0.308
Random Forest Classifier	0.894	0.798	0.195	0.594	0.292	0.250	0.296
Extreme Gradient Boosting	0.888	0.781	0.254	0.503	0.337	0.283	0.303
Extra Trees Classifier	0.884	0.774	0.168	0.465	0.244	0.196	0.227
Multilayer Perceptron Classifier	0.888	0.774	0.231	0.511	0.314	0.263	0.289
Naive Bayes	0.813	0.752	0.361	0.260	0.301	0.196	0.200
Quadratic Discriminant Analysis	0.805	0.748	0.369	0.251	0.298	0.190	0.195
K Neighbours Classifier	0.889	0.722	0.195	0.507	0.281	0.233	0.265
Supportive Vector Machine–Radial Kernel	0.891	0.692	0.035	0.829	0.067	0.058	0.152
Decision Tree Classifier	0.841	0.620	0.335	0.307	0.319	0.230	0.231
Dummy Classifier	0.888	0.500	0.000	0.000	0.000	0.000	0.000
Supportive Vector Machine–Linear Kernel^b^	0.890	0.000	0.035	0.532	0.062	0.052	0.110
Ridge Classifier^b^	0.892	0.000	0.060	0.726	0.111	0.095	0.187
Test set 1 (no./events = 3499/372)							
Gradient Boosting Classifier (PT = 0.500)	0.901	0.831	0.212	0.590	0.312	0.271	0.313
Discovery set (no./events = 8162/916)^a^							
Gradient Boosting Classifier (PT = 0.258)	0.880	0.822	0.434	0.464	0.447	0.380	0.381
Test set 1 (no./events = 3499/372)							
Gradient Boosting Classifier (PT = 0.258)	0.889	0.831	0.441	0.475	0.458	0.396	0.396
Test set 2 (no./events = 9403/1091)							
Gradient Boosting Classifier (PT = 0.258)	0.868	0.833	0.479	0.436	0.457	0.381	0.382
Test set 3 (no./events = 10 356/1504)							
Gradient Boosting Classifier (PT = 0.258)	0.855	0.852	0.553	0.502	0.526	0.441	0.442

Abbreviations: AUC, area under the curve; CI, confidence interval; MCC, the Matthews correlation coefficient; PT, probability threshold to indicate the positive class.

^a^ Metrics in the discovery sets are corrected with 10 iterations of 10‐fold cross‐validation.

^b^ Probability prediction is not supported for these models thus AUCs are not calculated.

### 3.5 Feature Selection

Feature selection was conducted using the RFECV method based on the weight F1 score. A total of 11 features were selected (score = 0.878), as shown in Figure S2. Subsequently, we retrained the ML models using these selected 11 features. The cross‐validation and test results are shown in Table S5. The GBC model remained the optimal model among all algorithms, with its hyperparameters detailed in Table S6. Delong's test revealed that the feature selection resulted in a statistically significant decline in performance in test set 1 (AUC: 0.825 vs. 0.831, *p* = 0.006). Because of our performance‐first strategy, we opted not to proceed with feature selection and maintained the original GBC model without feature selection for future applications.

### 3.6 Threshold Optimization

**FIGURE 2:**
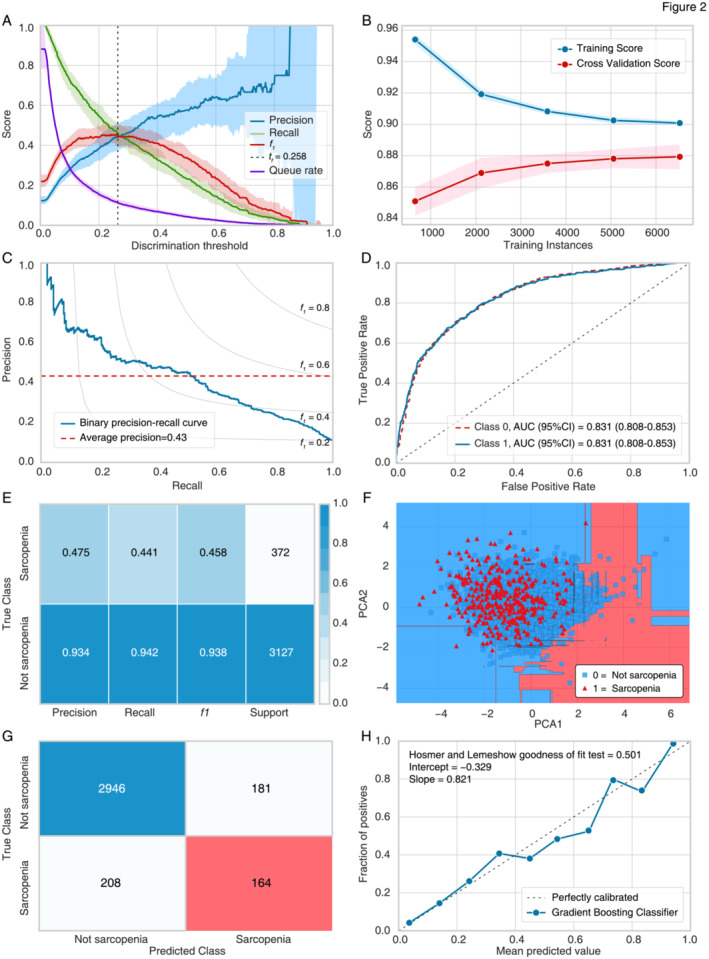
Threshold optimization, training and evaluation of the gradient boosting classifier (GBC) model. AUC, area under the curve; CI, confidence interval. (A) Discrimination threshold plot. (B) Training curve. (C) Precision–recall curve. (D) Receiver operating characteristic curve. (E) Classification report. (F) Decision boundary plot. (G) Confusion matrix. (H) Calibration curve.

The discrimination threshold plot demonstrated that setting the prediction threshold to 0.258 for the positive class achieved a favourable trade‐off between precision, recall, and F1 score (Figure 2A). The performance of the GBC model with the optimized threshold (*t* = 0.258) is summarized in Table 3. Overall, the GBC model exhibited improved classification performance in test set 1, with accuracy = 0.889, AUC = 0.831, recall = 0.441, precision = 0.475, F1 score = 0.458, Kappa = 0.396 and MCC = 0.396.

### 3.7 Model Assessment in Unseen Baseline Data

The learning curve of the GBC model in the training data is shown in Figure 2B. There was a noticeable trend towards convergence between the training and cross‐validation curves, suggesting effective learning of the classifier with increasing training instances. Subsequently, the GBC model underwent evaluation in test set 1. The precision–recall (PR) curve is shown in Figure 2C, with the area under the PR curve calculated at 0.43. The ROC curves showed that the AUC (95%CI) of the GBC model was 0.831 (0.808–0.853) (Figure 2D). The classification report of the GBC model is shown in Figure 2E, consistent with the results presented in Table 3. Moreover, the decision boundary of the GBC in the test data is demonstrated in Figure 2F. Analysis of the confusion matrix revealed that 2946 individuals (not sarcopenia) and 164 individuals (sarcopenia) were correctly predicted (Figure 2G). Additionally, the calibration curve of the GBC model demonstrated good consistency between model predictions and actual observations (Figure 2H). The Hosmer and Lemeshow goodness‐of‐fit test further corroborated these findings (*p* = 0.501).

### 3.8 Feature Importance and Model Explainability

The feature importance of the GBC model is evaluated using the model's built‐in feature importance ranking function, whereas model explainability was assessed using the SHAP method. The built‐in feature importance rankings of the GBC model are presented in Figures S3 and S4 for one‐hot encoded features and raw features, respectively. Consistently, the results highlight body weight, age and body height as the predominant features contributing to the classification power of the GBC model. Additionally, among the 20 FC items, lifting 5 kg emerges as the predominant feature.

**FIGURE 3:**
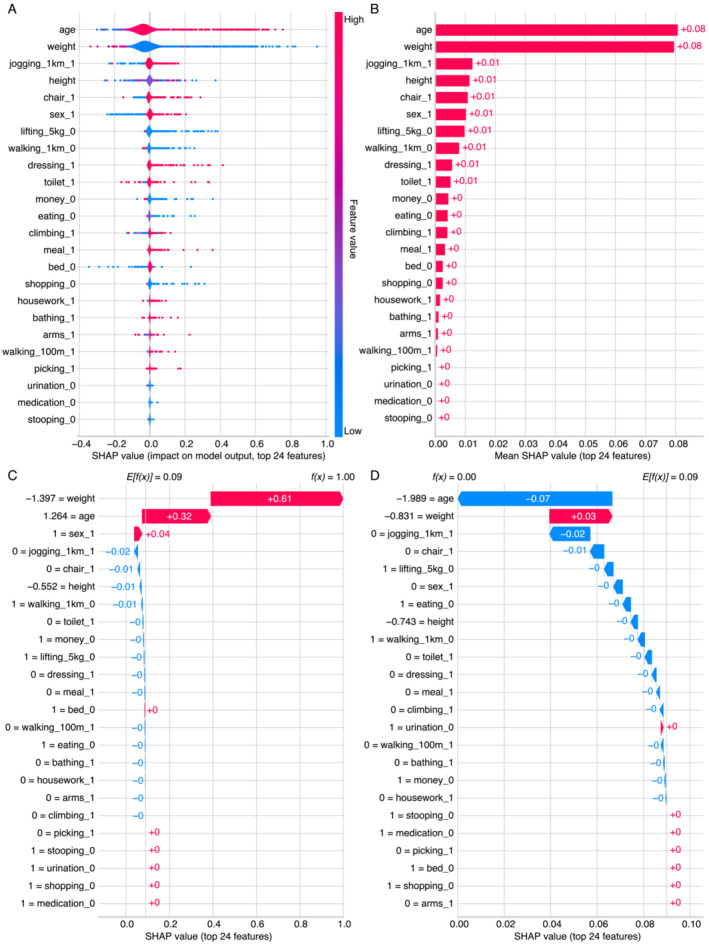
Model explainability using the SHapley Additive exPlanations (SHAP) method. (A) Group‐level explainability using a violin plot. (B) Group‐level explainability using a bar plot. (C) Individual‐level explainability (high probability). (D) Individual‐level explainability (low probability).

The results of the SHAP analysis are detailed in Figures 3 and S5. Initially, we evaluated the features in the GBC model that contributed to the prediction on a global level. The SHAP summary plot revealed that age, body weight and jogging 1 km were the top three contributors to the high likelihood of sarcopenia, with mean SHAP values of 0.08, 0.08 and 0.01, respectively (Figure 3A,B). We also examined the individual‐level risk predictions and their sources of risk specified by the SHAP values. For the patient with the highest predicted SHAP value (e.g., 1), body weight (+0.61), age (+0.32) and sex (+0.04) were the leading sources of risk contributing to the high SHAP value (Figures 3C and S5A). Conversely, for the patient with the lowest SHAP value (e.g., 0), age (−0.07), body weight (+0.03) and jogging 1 km (−0.02) were the primary sources of risk leading to the low SHAP value (Figures 3D and S5B).

### 3.9 Model Assessment in Follow‐Up Data

**FIGURE 4:**
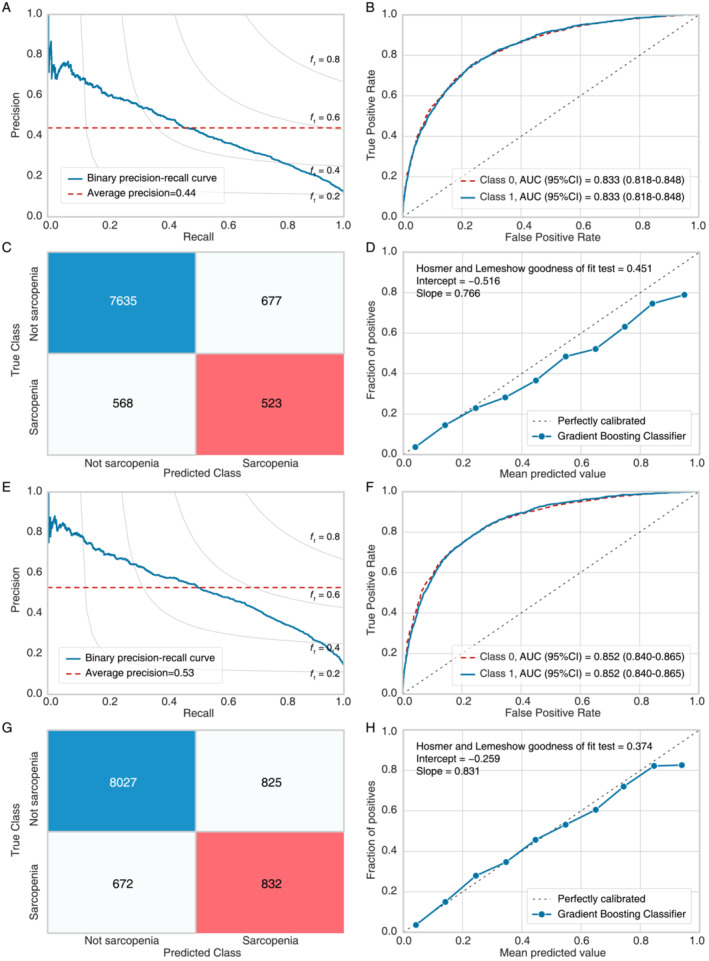
Model evaluation in longitudinal test datasets. AUC, area under the curve; CI, confidence interval. (A) Precision–recall curve of the gradient boosting classifier (GBC) model in test set 2. (B) Receiver operating characteristic (ROC) curve of the GBC model in test set 2. (C) Classification report of the GBC model in test set 2. (D) Calibration curve of the GBC model in test set 2. (E) Precision–recall curve of the GBC model in test set 3. (F) ROC curve of the GBC model in test set 3. (G) Classification report of the GBC model in test set 3. (H) Calibration curve of the GBC model in test set 3.

The GBC model was assessed in test set 2 (*n* = 9403) and test set 3 (*n* = 10 356) to assess its temporal reliability (Table 3 and Figure 4). In test set 2, the GBC model maintained its good performance, with an accuracy = 0.868, AUC = 0.833, recall = 0.479, precision = 0.436, F1 score = 0.457, Kappa = 0.381 and MCC = 0.382. The PR curve for test set 2 is displayed in Figure 4A, with an area under the PR curve of 0.44. The ROC curves indicated an AUC (95%CI) of 0.833 (0.818–0.848) for the GBC model (Figure 4B). The confusion matrix revealed accurate predictions for 7635 individuals without sarcopenia and 523 individuals with sarcopenia (Figure 4C). Furthermore, the calibration curve demonstrated good agreement between model predictions and actual observations (Figure 4D, Hosmer and Lemeshow goodness of fit test *p* = 0.501).

Similarly, the GBC model exhibited consistent performance in test set 3, with an accuracy = 0.855, AUC = 0.852, recall = 0.553, precision = 0.502, F1 score = 0.526, Kappa = 0.441 and MCC = 0.442. The PR curve (Figure 4E) showed an area under the PR curve of 0.53, whereas the ROC curves indicated an AUC (95%CI) of 0.852 (0.840–0.865) for the model (Figure 4F). The confusion matrix revealed accurate predictions for 8027 nonsarcopenic individuals and 832 individuals with sarcopenia (Figure 4G). Additionally, the calibration curve supported the favourable performance of the model (Hosmer and Lemeshow goodness‐of‐fit test *p* = 0.374, Figure 4H).

### 3.10 Subgroup Performance Analysis

The subgroup performance of the GBC model, stratified by age and sex, was evaluated across the three test sets (Table 4). Overall, the model maintained its strong performance across various subgroups investigated (all AUCs ≥ 0.750). Specifically, when considering the AUC as the reference metric for model performance, the model demonstrated relatively higher efficacy in older individuals and men, while exhibiting reduced efficacy in younger individuals and women.

**TABLE 4:** Model performance of different age and sex subgroups in the test sets.

Group	No./events	AUC (95%CI)	Kappa	Accuracy (95%CI)	Sensitivity	Specificity	PPV	NPV
Test set 1								
Overall	3499/372	0.831 (0.808–0.853)	0.396	0.889 (0.878, 0.899)	0.476	0.934	0.441	0.942
Age < 60 years	2160/91	0.750 (0.698–0.803)	0.098	0.951 (0.941, 0.960)	0.077	0.989	0.241	0.961
Age ≥ 60 years	1339/281	0.780 (0.750–0.811)	0.391	0.789 (0.766, 0.810)	0.559	0.850	0.497	0.879
Sex, men	1711/173	0.862 (0.834–0.890)	0.455	0.903 (0.888, 0.917)	0.497	0.949	0.521	0.944
Sex, women	1788/199	0.800 (0.766–0.833)	0.342	0.875 (0.859, 0.890)	0.392	0.936	0.433	0.925
Test set 2								
Overall	9403/1091	0.833 (0.821–0.846)	0.381	0.868 (0.861, 0.874)	0.479	0.919	0.436	0.931
Age < 60 years	4818/205	0.775 (0.742–0.807)	0.160	0.950 (0.943, 0.956)	0.132	0.986	0.293	0.962
Age ≥ 60 years	4585/886	0.789 (0.773–0.805)	0.360	0.782 (0.769, 0.793)	0.560	0.835	0.448	0.888
Sex, men	4491/486	0.852 (0.835–0.869)	0.392	0.880 (0.870, 0.889)	0.473	0.929	0.447	0.936
Sex, women	4912/605	0.817 (0.799–0.835)	0.372	0.857 (0.847, 0.866)	0.484	0.909	0.428	0.926
Test set 3								
Overall	10 356/1504	0.852 (0.842–0.862)	0.441	0.855 (0.849, 0.862)	0.553	0.907	0.502	0.923
Age < 60 years	4423/209	0.817 (0.785–0.849)	0.248	0.945 (0.938, 0.956)	0.220	0.981	0.365	0.962
Age ≥ 60 years	5933/1295	0.813 (0.800–0.826)	0.419	0.789 (0.778, 0.799)	0.607	0.839	0.513	0.884
Sex, men	4925/671	0.866 (0.852–0.881)	0.473	0.876 (0.866, 0.885)	0.547	0.927	0.543	0.928
Sex, women	5431/833	0.839 (0.825–0.853)	0.416	0.837 (0.827, 0.847)	0.558	0.888	0.474	0.917

Abbreviations: AUC, area under curve; CI, confidence interval; NPV, negative predictive value; PPV, positive predictive value.

### 3.11 Clinical Usefulness

DCA analysis was independently performed in the three test sets. The results were consistent, showing that if the threshold probability of an individual was > 0.02, using the model to predict the probability of sarcopenia adds more benefits than either the treat‐all‐subjects scheme or the treat‐none scheme (Figure S6A–C).

### 3.12 Model Deployment

To enable timely individualized application, we deployed the GBC model as an online application (https://xinqiaohospital‐don‐lyy‐sarco.streamlit.app), supporting both individual and batch predictions. The application provides outputs of both the predicted class and corresponding probability. The graphical user interface of this application is presented in Figure S7. Furthermore, the serialized model object that supports future reuse and the associated application code and files are publicly accessible in our GitHub repository (https://github.com/kevinlyy/sarco).

## 4 Discussion

This retrospective study involved 11 661 middle‐aged and older adults from multiple centres in China. The study aimed to address a significant real‐world public health and clinical challenge: evaluating the risk of sarcopenia through a test‐free approach applicable to nearly all scenarios. To our knowledge, this is the first large‐scale study that addresses this challenge using AI approaches based on features that are easy to obtain. Our findings have the potential to support public health professionals and clinicians in making informed decisions regarding the screening and surveillance of individuals at risk, thereby guiding management strategies to enhance outcomes.

The prevalence of sarcopenia in the baseline population, test set 2 and test set 3 were 11.0%, 11.6% and 14.5%, respectively (Table S3). Compared with other studies conducted in Asian populations, the prevalence of sarcopenia in the present study is consistent with most of them [2, 8, 31], whereas being higher [32] or lower [33] than others. Variations in the prevalence of sarcopenia may stem from differences in factors such as diagnostic criteria, region and age. In terms of age, we included both middle‐aged and older adults to maximize statistical power in the present study. Several reasons support this approach: Firstly, the study referenced by the AWGS 2019 to define sarcopenia‐related cut‐offs did not exclusively involve older patients. For example, the cut‐offs used to determine a low ASMI were based on a study including Japanese adults aged 40–89 years [34]. Secondly, the HGS thresholds have been widely used to define low HGS and/or sarcopenia in studies including younger adults [10], such as those aged > 35 years or 21–90 years [35, 36]. Thirdly, there is an emerging opinion that the conceptual definition of sarcopenia should not vary by age or condition [1], further justifying our approach.

Another interesting aspect regarding age is that the model appeared to perform better in older adults (≥ 60 years vs. < 60 years; Table 4). One possible explanation is that because of the higher prevalence of sarcopenia among the elderly [2, 3], the learner was trained with a greater number of instances from elderly individuals, resulting in better fitting within this subgroup. In support of this, the model also seemed to perform better in males, likely because of the higher incidence of sarcopenia in men than in women. In exploratory analyses (data not shown), we attempted to balance the distribution of age and positive instances in the training set using synthetic minority oversampling technique (SMOTE) and random under‐sampling (RUS). However, this did not yield significant improvements in model performance on the test set, suggesting that other underlying mechanisms are involved in this phenomenon. Future research should incorporate data from more individuals with sarcopenia, particularly including more middle‐aged individuals with sarcopenia, to further analyse this issue.

Despite the primary objective of developing an AI model for identifying sarcopenia, the present study also provided another intriguing finding: the comparison of different FC items in predicting the occurrence of sarcopenia. We provided several lines of evidence to elucidate the relative importance of the 20 FC items, including AUCs (Table 2), the model's built‐in feature importance ranking (Figures S3–S4) and the SHAP results (Figures 3 and S5). These findings offer insights into at least two aspects: Firstly, quantifying the importance of various FC items can assist public health experts and clinicians in decision‐making by focusing on those items that more strongly indicate the onset of sarcopenia. When individuals/patients exhibit abnormalities in these key indicators, further examinations may be necessary to confirm diagnosis or initiate timely interventions. Importantly, the simplicity of these items enables self‐assessment, thereby reducing the workload of healthcare professionals. Secondly, our findings suggest that FC items related to lower limb muscle strength/function (e.g., jogging 1 km and walking 1 km) may hold significant value for identifying sarcopenia. However, popular diagnostic frameworks lack assessment regarding lower limb muscle strength/function. For instance, the EWGSOP2 and AWGS 2019 only assess HGS to reflect the whole spectrum of muscle strength [2, 3]. Recently, a consensus‐based conceptual definition of sarcopenia proposed by all relevant scientific societies worldwide, namely, the Global Leadership Initiative in Sarcopenia (GLIS), has introduced muscle‐specific strength as part of the conceptual definition of sarcopenia [1]. Muscle‐specific strength, defined as strength standardized to muscle size, includes assessments such as leg extension maximal strength standardized to quadriceps muscle volume [37]. Similarly, another study also found that besides reduced muscle mass, impairments in the functional performance of lower limbs are more significant indicators of sarcopenia than loss of muscle strength [38]. These lines of evidence align well with the observations in our study, suggesting that assessment of lower limb muscle strength/function should be an essential component of sarcopenia diagnosis. Future research directions could involve further standardizing protocols for lower limb strength measurement and establishing cut‐off points for different ethnicities and populations.

A recent study proposed a linear framework‐based prediction model for identifying sarcopenia in older Chinese adults [39]. This study used sex, BMI, mean systolic blood pressure, mean diastolic blood pressure and pain as input features, reporting an AUC (95%CI) of 0.76 (95%CI = 0.73–0.79). Compared with the referenced study, our research exhibits several distinct strengths: Firstly, our study is based on a more stringent scientific hypothesis, and the variables utilized in our model, such as ADLs and IADLs, have widely accepted biological associations with sarcopenia, as recognized in the latest GLIS consensus endorsed by most domain experts worldwide [1]. Consequently, the model proposed in our study is readily understandable and interpretable in real‐world settings. In contrast, there is currently insufficient evidence supporting blood pressure changes as either causes or outcomes of sarcopenia. Secondly, we employed a larger sample size comprising a more diverse population (middle‐aged and older adults) and achieved superior model performance (AUC of 0.831, 0.833 and 0.852 in the three test sets, compared with 0.76 in the referenced study). Thirdly, the tree‐based nature of the GBC model we utilized can handle non‐linear correlations. Fourthly, we tested the model in both cross‐sectional and longitudinal datasets, thereby enhancing the credibility of our results. Most importantly, our model was test‐free and deployed as a publicly accessible web application, greatly facilitating its application across various scenarios.

There are several potential limitations of this study that must be noted. First, the ASMI in the present study was derived from an anthropometric equation validated for use in Chinese [26]. DEXA or BIA might provide a more accurate measurement of the skeletal muscle mass to diagnose sarcopenia [2]. However, the equation has good consistency with dual‐energy X‐ray imaging, and the variables in the equation are simple to obtain, which should increase the usability of the study parameters in broader settings. Nevertheless, future studies using more advanced technologies to measure skeletal muscle mass are needed to replicate our study. Second, because Asian populations have anthropometric differences compared with their Western counterparts [2], these results should be re‐evaluated when applied in non‐Asian populations. Third, the choice of input features was primarily based on our study hypothesis and was limited to those parameters closely related to the diagnostic criteria of sarcopenia. Other unanalysed parameters might help increase the model's performance. Fourth, although the DCA supports the clinical usefulness of our model, further evaluation in individuals/patients with specific intervention data is imperative to confirm this finding. Individuals with model‐predicted sarcopenia are recommended to undergo further formal diagnosis of sarcopenia. Finally, the diagnosis of sarcopenia was performed retrospectively, so the risk of misclassification cannot be completely eliminated. However, we included only individuals with complete data. Previous studies have also suggested that our approach is feasible [39]. Nevertheless, prospective studies using predefined sarcopenia data would help further validate the robustness of our results. Future studies are needed to address the above issues.

In conclusion, based on a hypothesis, we developed and comprehensively validated an ML model to predict sarcopenia in middle‐aged and older adults. The model used a unique test‐free design, showing good performance for predicting sarcopenia in cross‐sectional and longitudinal validation data. Importantly, the model was deployed as a web‐based application and exported as a serialized file to provide flexible options for different application scenarios. Together, these findings might assist public health professionals or clinicians with decision‐making to help guide management strategies to optimize the screening and surveillance of sarcopenia.

## Ethics Statement

The authors certify that the ethical guidelines for publishing of the *Journal of Cachexia, Sarcopenia and Muscle*: update 2019 have been followed [40]. National and international research ethics guidelines were followed, including the Deontological Code of Ethics and the 1964 Declaration of Helsinki and its later amendments. All patients provided written consent for the use of their data, and the study protocol of the CHARLS was approved by the Ethical Review Committee of Peking University (approval number: IRB00001052‐11015).

## Conflicts of Interest

The authors declare no conflicts of interest.

## Supporting information


**Table S1.** Functional capacity indices included for analysis in the present study
**Table S2.** Machine learning algorithms used in the present study
**Table S3.** Features used for modelling and their distribution in the discovery and test sets
**Table S4.** Hyper‐parameters of the gradient boosting classifier
**Table S5.** Model development and validation using selected features
**Table S6.** Hyper‐parameters of the gradient boosting classifier with selected input features
**Figure S1.** A flowchart of the subject inclusion.
**Figure S2.** Recursive feature elimination for feature selection
**Figure S3.** Model's built‐in feature importance by one‐hot encoded features
**Figure S4.** Model's built‐in feature importance by raw features
**Figure S5.** Force plot of the SHapley Additive exPlanations (SHAP) analysis. (A)
Explainability at the individual level (high probability). (B) Explainability at the individual level (low probability).
**Figure S6.** Decision curve analysis (DCA) in the three test sets. (A) DCA in test set 1 (*n* = 3499). (B) DCA in test set 2 (*n* = 9403). (C) DCA in test set 3 (*n* = 10 356).
**Figure S7.** User interface of the AITIS model
