# Supplementary material for: An Artificial Intelligence Approach for Test‐Free Identification of Sarcopenia
Source: J Cachexia Sarcopenia Muscle. 2024 Nov 8;15(6):2765–80. doi: 10.1002/jcsm.13627 (PMC11634523; doi:10.1002/jcsm.13627)
Supplement: Supplementary file 1 — Table S1. Functional capacity indices included for analysis in the present study Table S2. Machine learning algorithms used in the present study Table S3. Features used for modelling and their distribution in the discovery and test sets Table S4. Hyper‐parameters of the gradient boosting classifier Table S5. Model development and validation using selected features Table S6. Hyper‐parameters of the gradient boosting classifier with selected input features Figure S1. A flowchart of the subject inclusion. Figure S2. Recursive feature elimination for feature selection Figure S3. Model's built‐in feature importance by one‐hot encoded features Figure S4. Model's built‐in feature importance by raw features Figure S5. Force plot of the SHapley Additive exPlanations (SHAP) analysis. (A) Explainability at the individual level (high probability). (B) Explainability at the individual level (low probability). Figure S6. Decision curve analysis (DCA) in the three test sets. (A) DCA in test set 1 (n = 3499). (B) DCA in test set 2 (n = 9403). (C) DCA in test set 3 (n = 10 356). Figure S7. User interface of the AITIS model [file JCSM-15-2765-s001.pdf]

Title: An artificial intelligence approach for test-free identification of sarcopenia

Supplementary files

Number	Description
1	**Table S1.** Functional capacity items included for analysis in the present study
2	**Table S2.** Machine learning algorithms used in the present study
3	**Table S3.** Features used for modelling and their distribution in the discovery and test sets
4	**Table S4.** Hyper-parameters of the gradient boosting classifier
5	**Table S5.** Model development and validation using selected features
6	**Table S6.** Hyper-parameters of the gradient boosting classifier with selected input features
7	**Figure S1.** A flowchart of the subject inclusion.
8	**Figure S2.** Recursive feature elimination for feature selection
9	**Figure S3.** Model's built-in feature importance by one-hot encoded features
10	**Figure S4.** Model's built-in feature importance by raw features
11	**Figure S5.** Force plot of the SHapley Additive exPlanations (SHAP) analysis. (A) Explainability at the individual level (high probability). (B) Explainability at the individual level (low probability).
12	**Figure S6.** Decision curve analysis (DCA) in the three test sets. (A) DCA in test set 1 (n=3499). (B) DCA in test set 2 (n=9403). (C) DCA in test set 3 (n=10356).
13	**Figure S7.** User interface of the AITIS model

Table S1. Functional capacity indices included for analysis in the present study

Category	Index	Detailed description
ADL	Dressing	Do you have some difficulty with dressing?
	Bathing	Do you have some difficulty with bathing?
	Eating	Do you have some difficulty with eating?
	Bed	Do you have some difficulty with getting in and out of bed?
	Toilet	Do you have some difficulty with using the toilet?
	Urination	Do you have some difficulty with controlling urination and defecation?
IADL	Money	Do you have some difficulty with managing money?
	Medication	Do you have some difficulty with taking medications?
	Shopping	Do you have some difficulty with shopping for groceries?
	Meal	Do you have some difficulty with preparing meals?
	Housework	Do you have some difficulty with cleaning house?
Other	Jogging 1km	Do you have some difficulty with running or jogging 1km?
	Walking 1km	Do you have some difficulty with walking 1km?
	Walking 100m	Do you have some difficulty with walking 100m?
	Chair	Do you have some difficulty with getting up from a chair after sitting for long periods?
	Climbing	Do you have some difficulty with climbing several flights of stairs without resting?
	Stooping	Do you have some difficulty with stooping, kneeling, or crouching?
	Lifting 5kg	Do you have some difficulty with lifting or carrying weights over 5kg?
	Picking	Do you have some difficulty with picking up a coin from the table?
	Arm	Do you have some difficulty with reaching arms above shoulder level?

Abbreviations: ADL, activities of daily living; IADL, instrumental activities of daily living.

Table S2. Machine learning algorithms used in the present study

Algorithm name	Framework	Version	Class/Module	Hyper-parameters (default)
Gradient Boosting Classifier	scikit-learn	0.23.2	GradientBoostingClassifier	ccp_alpha=0.0, criterion=friedman_mse, init=None, learning_rate=0.1, loss=deviance, max_depth=3, max_features=None, max_leaf_nodes=None, min_impurity_decrease=0.0, min_impurity_split=None, min_samples_leaf=1, min_samples_split=2, min_weight_fraction_leaf=0.0, n_estimators=100, n_iter_no_change=None, presort=deprecated, random_state=123, subsample=1.0, tol=0.0001, validation_fraction=0.1, verbose=0, warm_start=False
Ada Boost Classifier	scikit-learn	0.23.2	AdaBoostClassifier	algorithm=SAMME.R, base_estimator=None, learning_rate=1.0, n_estimators=50, random_state=123
Logistic Regression	scikit-learn	0.23.2	LogisticRegression	C=1.0, class_weight=None, dual=False, fit_intercept=True, intercept_scaling=1, l1_ratio=None, max_iter=1000, multi_class=auto, n_jobs=None, penalty=l2, random_state=123, solver=lbfgs, tol=0.0001, verbose=0, warm_start=False
Linear Discriminant Analysis	scikit-learn	0.23.2	LinearDiscriminantAnalysis	n_components=None, priors=None, shrinkage=None, solver=svd, store_covariance=False, tol=0.0001
Light Gradient Boosting Machine	lightgbm	3.3.2	lightgbm	boosting_type=gbdt, class_weight=None, colsample_bytree=1.0, importance_type=split, learning_rate=0.1, max_depth=-1, min_child_samples=20, min_child_weight=0.001, min_split_gain=0.0, n_estimators=100, n_jobs=-1, num_leaves=31, objective=None, random_state=123, reg_alpha=0.0, reg_lambda=0.0, silent=warn, subsample=1.0, subsample_for_bin=200000, subsample_freq=0
Random Forest Classifier	scikit-learn	0.23.2	RandomForestClassifier	bootstrap=True, ccp_alpha=0.0, class_weight=None, criterion=gini, max_depth=None, max_features=auto, max_leaf_nodes=None, max_samples=None, min_impurity_decrease=0.0, min_impurity_split=None, min_samples_leaf=1, min_samples_split=2, min_weight_fraction_leaf=0.0, n_estimators=100, n_jobs=-1, oob_score=False, random_state=123, verbose=0, warm_start=False
Extreme Gradient Boosting	xgboost	2.1.0	XGBClassifier	objective=binary:logistic, use_label_encoder=False, base_score=None, booster=gbtree, callbacks=None, colsample_bylevel=None, colsample_bynode=None, colsample_bytree=None, early_stopping_rounds=None, enable_categorical=False, eval_metric=None, gamma=None, gpu_id=None, grow_policy=None, importance_type=None, interaction_constraints=None, learning_rate=None, max_bin=None, max_cat_to_onehot=None, max_delta_step=None, max_depth=None, max_leaves=None, min_child_weight=None, missing=nan, monotone_constraints=None, n_estimators=100, n_jobs=-1, num_parallel_tree=None, predictor=None, random_state=123, reg_alpha=None, reg_lambda=None, sampling_method=None, scale_pos_weight=None, subsample=None, tree_method=auto, validate_parameters=None, verbosity=0

Extra Trees Classifier	scikit-learn	0.23.2	ExtraTreesClassifier	bootstrap=False, ccp_alpha=0.0, class_weight=None, criterion=gini, max_depth=None, max_features=auto, max_leaf_nodes=None, max_samples=None, min_impurity_decrease=0.0, min_impurity_split=None, min_samples_leaf=1, min_samples_split=2, min_weight_fraction_leaf=0.0, n_estimators=100, n_jobs=-1, oob_score=False, random_state=123, verbose=0, warm_start=False
Multilayer Perceptron Classifier	scikit-learn	0.23.2	MLPClassifier	activation=relu, alpha=0.0001, batch_size=auto, beta_1=0.9, beta_2=0.999, early_stopping=False, epsilon=1e-08, hidden_layer_sizes=(100,), learning_rate=constant, learning_rate_init=0.001, max_fun=15000, max_iter=500, momentum=0.9, n_iter_no_change=10, nesterovs_momentum=True, power_t=0.5, random_state=123, shuffle=True, solver=adam, tol=0.0001, validation_fraction=0.1, verbose=False, warm_start=False
Naive Bayes	scikit-learn	0.23.2	GaussianNB	priors=None, var_smoothing=1e-09
Quadratic Discriminant Analysis	scikit-learn	0.23.2	QuadraticDiscriminantAnalysis	priors=None, reg_param=0.0, store_covariance=False, tol=0.0001
K Neighbors Classifier	scikit-learn	0.23.2	KNeighborsClassifier	algorithm=auto, leaf_size=30, metric=minkowski, metric_params=None, n_jobs=-1, n_neighbors=5, p=2, weights=uniform
Supportive Vector Machine - Radial Kernel	scikit-learn	0.23.2	SVC	C=1.0, break_ties=False, cache_size=200, class_weight=None, coef0=0.0, decision_function_shape=ovr, degree=3, gamma=auto, kernel=rbf, max_iter=-1, probability=True, random_state=123, shrinking=True, tol=0.001, verbose=False
Decision Tree Classifier	scikit-learn	0.23.2	DecisionTreeClassifier	ccp_alpha=0.0, class_weight=None, criterion=gini, max_depth=None, max_features=None, max_leaf_nodes=None, min_impurity_decrease=0.0, min_impurity_split=None, min_samples_leaf=1, min_samples_split=2, min_weight_fraction_leaf=0.0, presort=deprecated, random_state=123, splitter=best
Dummy Classifier	scikit-learn	0.23.2	DummyClassifier	constant=None, random_state=123, strategy=prior
Supportive Vector Machine - Linear Kernel	scikit-learn	0.23.2	SVC	alpha=0.0001, average=False, class_weight=None, early_stopping=False, epsilon=0.1, eta0=0.001, fit_intercept=True, l1_ratio=0.15, learning_rate=optimal, loss=hinge, max_iter=1000, n_iter_no_change=5, n_jobs=-1, penalty=l2, power_t=0.5, random_state=123, shuffle=True, tol=0.001, validation_fraction=0.1, verbose=0, warm_start=False
Ridge Classifier	scikit-learn	0.23.2	RidgeClassifier	alpha=1.0, class_weight=None, copy_X=True, fit_intercept=True, max_iter=None, normalize=False, random_state=123, solver=auto, tol=0.001

Table S3. Features used for modelling and their distribution in the discovery and test sets

	Baseline data				Follow-up data	
Features	Overall (n=11661)	Discovery set (n=3499)	Test set 1 (n=8162)	*P*	Test set 2 (n=9403)	Test set 3 (n=10356)
Age, years	57.0 [50.0, 64.0][1]	57.0 [50.0, 63.0]	57.0 [50.0, 64.0]	0.004	59.0 [53.0, 66.0]	61.0 [55.0, 67.0]
Sex, men	5634 (48.3)[2]	1711 (48.9)	3923 (48.1)	0.420	4491 (47.8)	4925 (47.6)
Height, m	1.6 [1.5, 1.6]	1.6 [1.5, 1.6]	1.6 [1.5, 1.6]	0.081	1.6 [1.5, 1.6]	1.6 [1.5, 1.6]
Weight, kg	58.0 [51.2, 65.8]	58.0 [51.4, 66.0]	58.0 [51.2, 65.7]	0.297	58.8 [51.8, 66.7]	58.4 [51.6, 66.6]
Dressing	321 (2.8)	100 (2.9)	221 (2.7)	0.694	320 (3.4)	496 (4.8)
Bathing	360 (3.1)	110 (3.1)	250 (3.1)	0.863	381 (4.1)	596 (5.8)
Eating	135 (1.2)	39 (1.1)	96 (1.2)	0.849	122 (1.3)	192 (1.9)
Bed	312 (2.7)	100 (2.9)	212 (2.6)	0.461	359 (3.8)	629 (6.1)
Toilet	949 (8.1)	293 (8.4)	656 (8.0)	0.567	929 (9.9)	1310 (12.6)
Urination	335 (2.9)	106 (3.0)	229 (2.8)	0.547	298 (3.2)	407 (3.9)
Money	1153 (9.9)	346 (9.9)	807 (9.9)	1.000	815 (8.7)	1056 (10.2)
Medication	570 (4.9)	168 (4.8)	402 (4.9)	0.812	331 (3.5)	460 (4.4)
Shopping	622 (5.3)	191 (5.5)	431 (5.3)	0.728	517 (5.5)	721 (7.0)
Meal	543 (4.7)	161 (4.6)	382 (4.7)	0.891	549 (5.8)	773 (7.5)
Housework	601 (5.2)	173 (4.9)	428 (5.2)	0.532	716 (7.6)	1160 (11.2)
Jogging 1km	5542 (47.5)	1655 (47.3)	3887 (47.6)	0.764	4876 (51.9)	5512 (53.2)
Walking 1km	944 (8.1)	275 (7.9)	669 (8.2)	0.566	1249 (13.3)	1717 (16.6)
Walking 100m	158 (1.4)	54 (1.5)	104 (1.3)	0.287	315 (3.3)	609 (5.9)
Chair	2683 (23.0)	830 (23.7)	1853 (22.7)	0.241	2534 (26.9)	2946 (28.4)
Climbing	4185 (35.9)	1305 (37.3)	2880 (35.3)	0.040	3805 (40.5)	4386 (42.4)
Stooping	2936 (25.2)	903 (25.8)	2033 (24.9)	0.316	2928 (31.1)	3412 (32.9)
Lifting 5kg	852 (7.3)	252 (7.2)	600 (7.4)	0.807	1023 (10.9)	1330 (12.8)
Picking	264 (2.3)	81 (2.3)	183 (2.2)	0.862	285 (3.0)	404 (3.9)
Arm	881 (7.6)	264 (7.5)	617 (7.6)	1.000	875 (9.3)	1059 (10.2)
Sarcopenia	1288 (11.0)	372 (10.6)	916 (11.2)	0.368	1091 (11.6)	1504 (14.5)

[1] Median [interquartile range], all such values.

[2] Number (percentage), all such values.

Table S4. Hyper-parameters of the gradient boosting classifier

Parameters	Value
ccp_alpha	0
criterion	friedman_mse
init	None
learning_rate	0.1
loss	deviance
max_depth	3
max_features	None
max_leaf_nodes	None
min_impurity_decrease	0
min_impurity_split	None
min_samples_leaf	1
min_samples_split	2
min_weight_fraction_leaf	0
n_estimators	100
n_iter_no_change	None
random_state	123
subsample	1
tol	0.0001
validation_fraction	0.1
verbose	0
warm_start	FALSE
probability_threshold	0.285

Table S5. Model development and validation using selected features

Model	Accuracy	AUC	Recall	Precision	F1	Kappa	MCC
Discovery set (no./events=8162/916)[1]							
Gradient Boosting Classifier	0.895	0.822	0.229	0.591	0.328	0.283	0.322
Ada Boost Classifier	0.892	0.813	0.209	0.569	0.303	0.258	0.297
Logistic Regression	0.894	0.810	0.167	0.623	0.261	0.224	0.282
Linear Discriminant Analysis	0.888	0.809	0.212	0.523	0.298	0.249	0.281
Multilayer Perceptron Classifier	0.892	0.805	0.248	0.547	0.333	0.285	0.314
Light Gradient Boosting Machine	0.891	0.804	0.227	0.535	0.318	0.269	0.299
Random Forest Classifier	0.892	0.791	0.237	0.556	0.329	0.280	0.312
Extreme Gradient Boosting	0.889	0.789	0.249	0.513	0.333	0.281	0.304
Naive Bayes	0.817	0.777	0.406	0.283	0.333	0.231	0.237
Quadratic Discriminant Analysis	0.817	0.773	0.396	0.279	0.327	0.225	0.230
Extra Trees Classifier	0.886	0.769	0.231	0.493	0.312	0.259	0.282
K Neighbors Classifier	0.887	0.718	0.215	0.498	0.298	0.247	0.274
Supportive Vector Machine - Radial Kernel	0.894	0.696	0.087	0.719	0.154	0.133	0.223
Decision Tree Classifier	0.842	0.621	0.335	0.310	0.322	0.232	0.233
Dummy Classifier	0.888	0.500	0.000	0.000	0.000	0.000	0.000
Supportive Vector Machine - Linear Kernel[2]	0.888	0.000	0.007	0.150	0.013	0.010	0.026
Ridge Classifier[2]	0.892	0.000	0.056	0.729	0.103	0.089	0.180
Test set 1 (no./events=3499/372)							
Gradient Boosting Classifier (PT=0.500)	0.898	0.825	0.177	0.564	0.270	0.231	0.276

Abbreviations: AUC, area under the curve; CI, confidence interval; MCC, the Matthews correlation coefficient; PT, probability threshold to indicate the positive class.

[1] Metrics in the discovery sets are corrected with 10 iterations of 10-fold cross-validation.

[2] Probability prediction is not supported for these models thus AUCs are not calculated.

Table S6. Hyper-parameters of the gradient boosting classifier with selected input features

Parameters	Value
ccp_alpha	0
criterion	friedman_mse
init	None
learning_rate	0.1
loss	deviance
max_depth	3
max_features	None
max_leaf_nodes	None
min_impurity_decrease	0
min_impurity_split	None
min_samples_leaf	1
min_samples_split	2
min_weight_fraction_leaf	0
n_estimators	100
n_iter_no_change	None
random_state	123
subsample	1
tol	0.0001
validation_fraction	0.1
verbose	0
warm_start	FALSE
probability_threshold	0.26

Figure S1

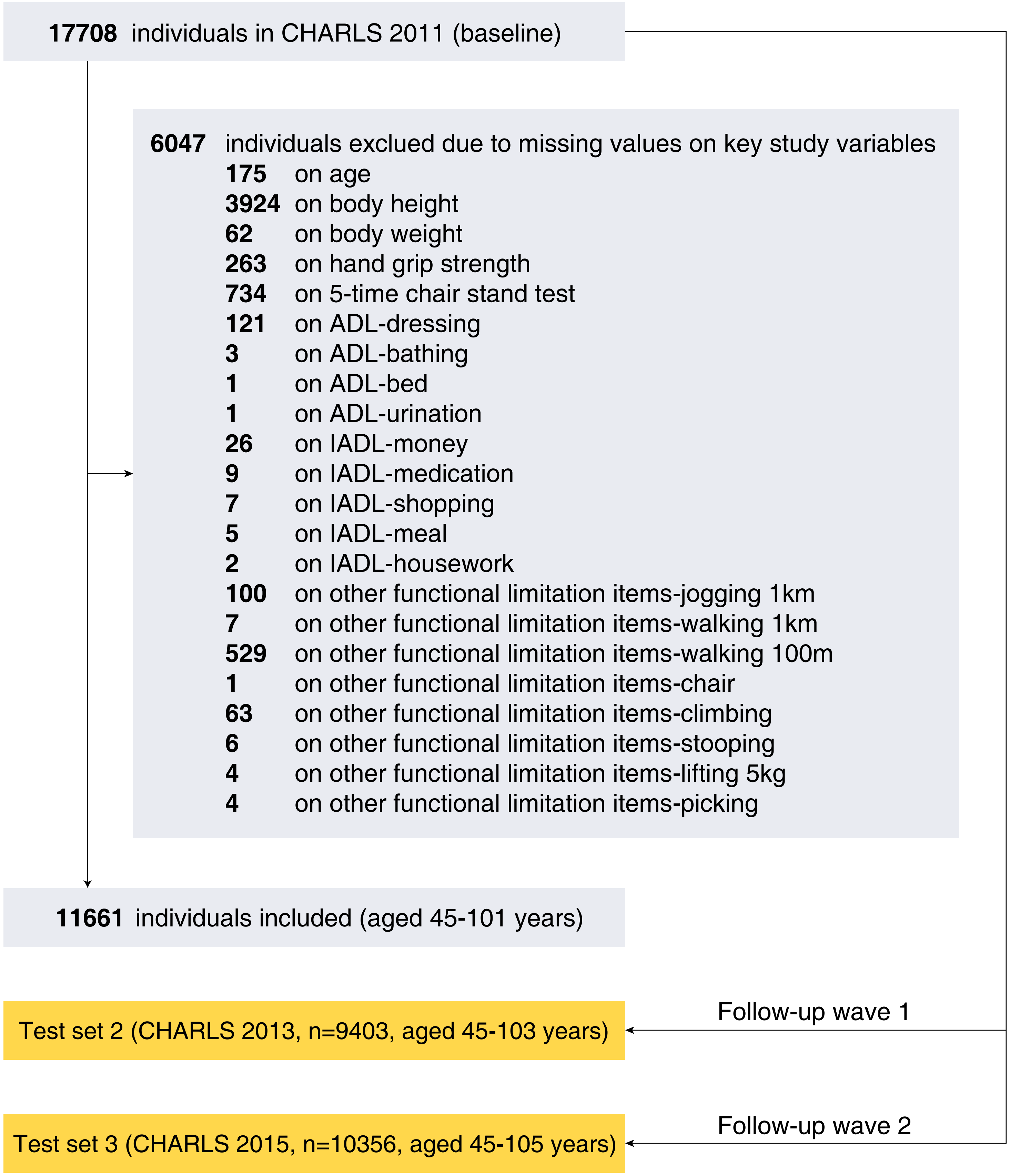
17708 individuals in CHARLS 2011 (baseline)
6047 individuals exclued due to missing values on key study variables
175 on age
3924 on body height
62 on body weight
263 on hand grip strength
734 on 5-time chair stand test
121 on ADL-dressing
3 on ADL-bathing
1 on ADL-bed
1 on ADL-urination
26 on IADL-money
9 on IADL-medication
7 on IADL-shopping
5 on IADL-meal
2 on IADL-housework
100 on other functional limitation items-jogging 1km
7 on other functional limitation items-walking 1km
529 on other functional limitation items-walking 100m
1 on other functional limitation items-chair
63 on other functional limitation items-climbing
6 on other functional limitation items-stooping
4 on other functional limitation items-lifting 5kg
4 on other functional limitation items-picking
11661 individuals included (aged 45-101 years)
Follow-up wave 1
Test set 2 (CHARLS 2013, n=9403, aged 45-103 years)
Follow-up wave 2
Test set 3 (CHARLS 2015, n=10356, aged 45-105 years)

Figure S2

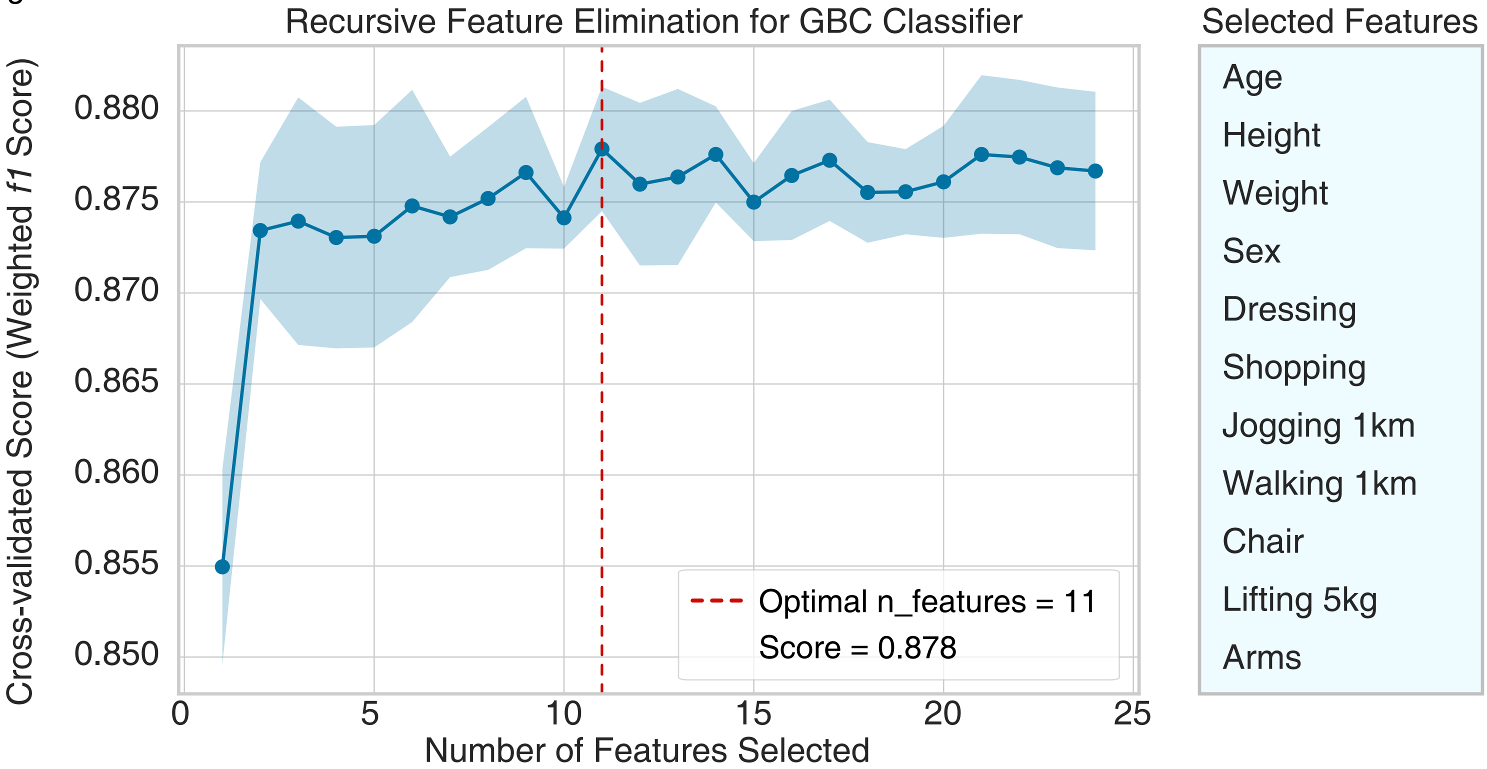

Recursive Feature Elimination for GBC Classifier
Cross-validated Score (Weighted f1 Score)
0.880
0.875
0.870
0.865
0.860
0.855
0.850
0
5
10
15
20
25
Number of Features Selected
Optimal n_features = 11
Score = 0.878
Selected Features
Age
Height
Weight
Sex
Dressing
Shopping
Jogging 1km
Walking 1km
Chair
Lifting 5kg
Arms

Figure S3

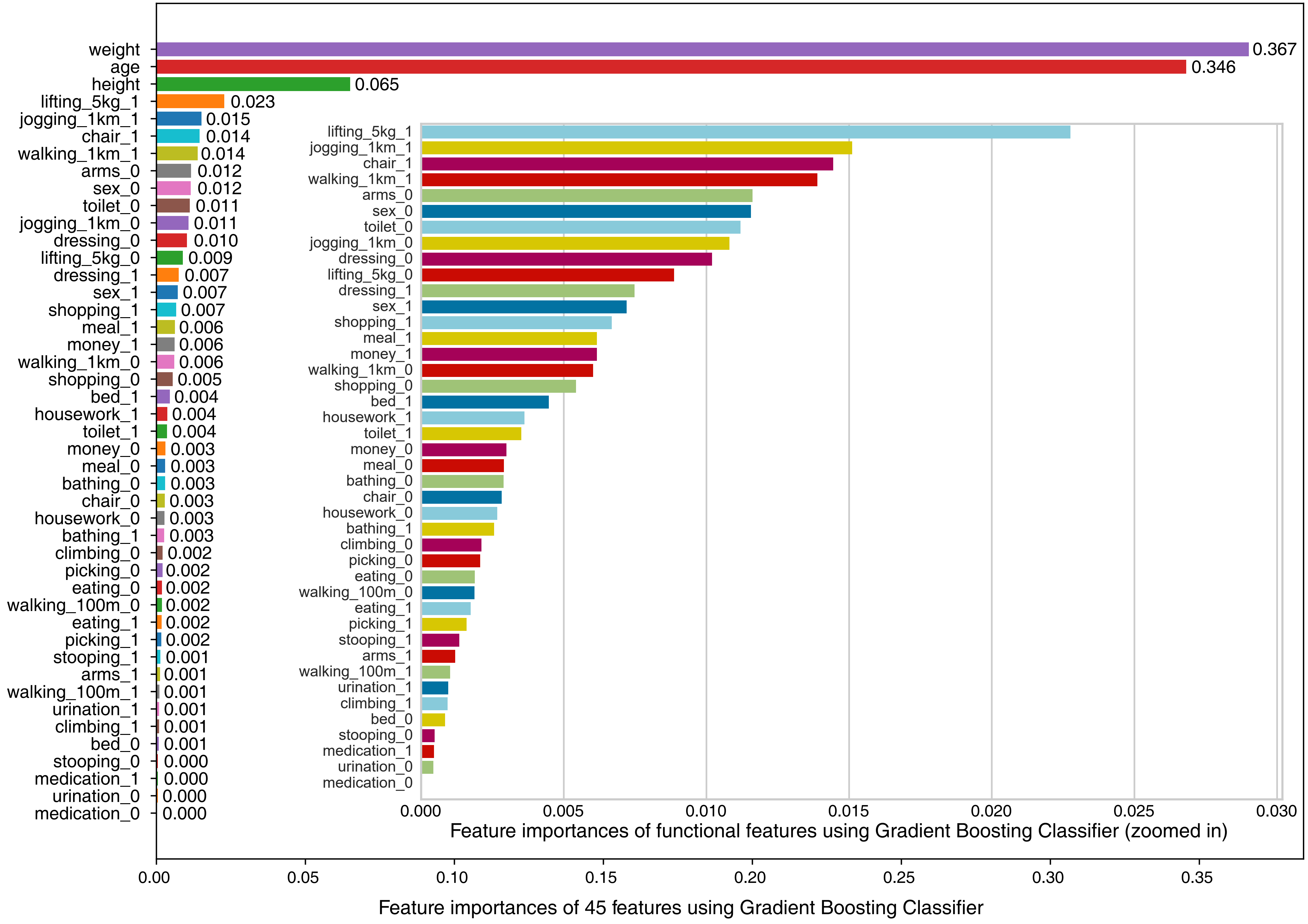
weight 0.367
age 0.346
height 0.065
lifting_5kg_1 0.023
jogging_1km_1 0.015
chair_1 0.014
walking_1km_1 0.014
arms_0 0.012
sex_0 0.012
toilet_0 0.011
jogging_1km_0 0.011
dressing_0 0.010
lifting_5kg_0 0.009
dressing_1 0.007
sex_1 0.007
shopping_1 0.007
meal_1 0.006
money_1 0.006
walking_1km_0 0.006
shopping_0 0.005
bed_1 0.004
housework_1 0.004
toilet_1 0.004
money_0 0.003
meal_0 0.003
bathing_0 0.003
chair_0 0.003
housework_0 0.003
bathing_1 0.003
climbing_0 0.002
picking_0 0.002
eating_0 0.002
walking_100m_0 0.002
eating_1 0.002
picking_1 0.002
stooping_1 0.001
arms_1 0.001
walking_100m_1 0.001
urination_1 0.001
climbing_1 0.001
bed_0 0.001
stooping_0 0.000
medication_1 0.000
urination_0 0.000
medication_0 0.000
0.00
0.05
0.10
0.15
0.20
0.25
0.30
0.35
Feature importances of 45 features using Gradient Boosting Classifier
0.000
0.005
0.010
0.015
0.020
0.025
0.030
Feature importances of functional features using Gradient Boosting Classifier (zoomed in)

Figure S4

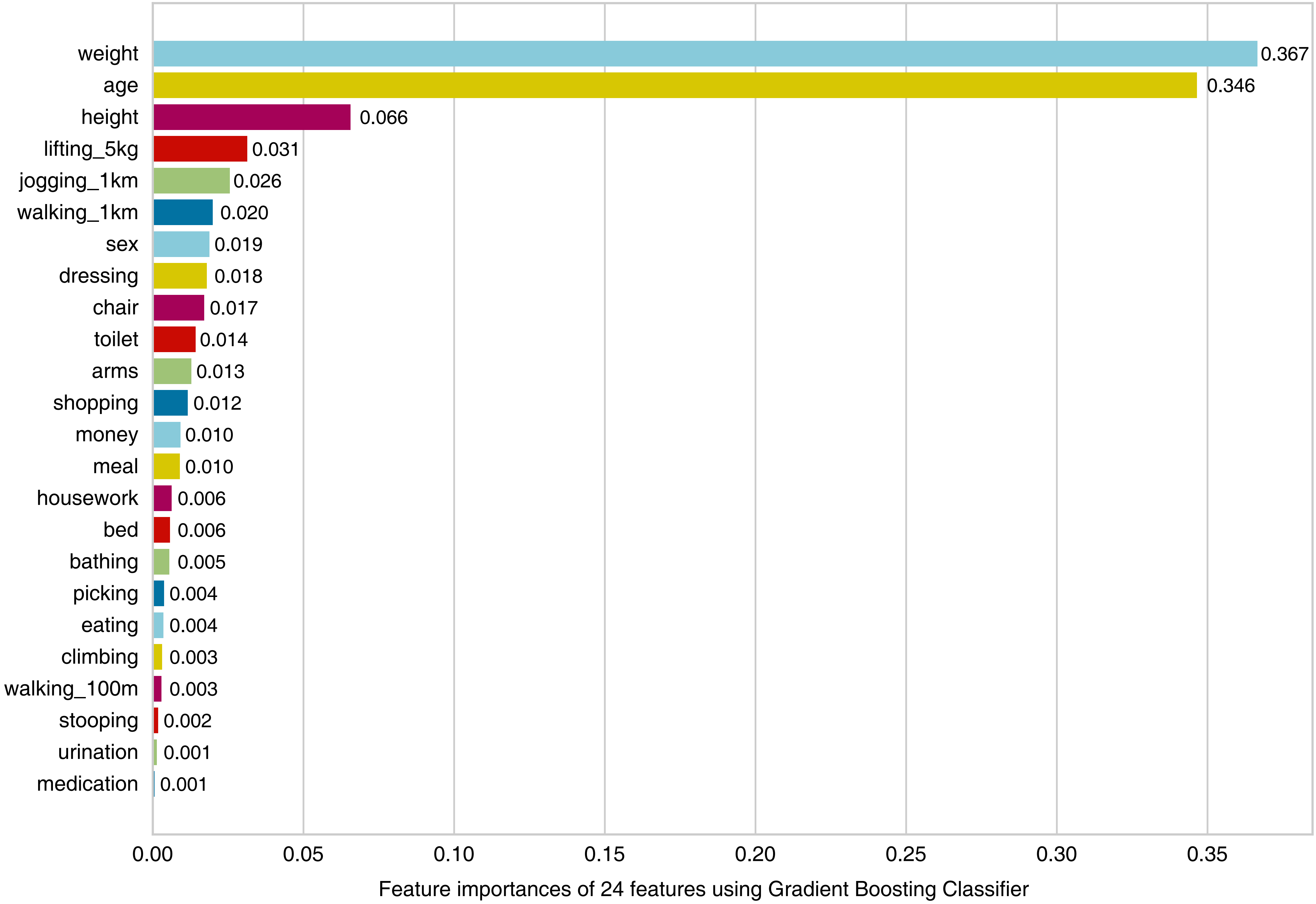
weight
0.367
age
0.346
height
0.066
lifting_5kg
0.031
jogging_1km
0.026
walking_1km
0.020
sex
0.019
dressing
0.018
chair
0.017
toilet
0.014
arms
0.013
shopping
0.012
money
0.010
meal
0.010
housework
0.006
bed
0.006
bathing
0.005
picking
0.004
eating
0.004
climbing
0.003
walking_100m
0.003
stooping
0.002
urination
0.001
medication
0.001
0.00
0.05
0.10
0.15
0.20
0.25
0.30
0.35
Feature importances of 24 features using Gradient Boosting Classifier

Figure S5

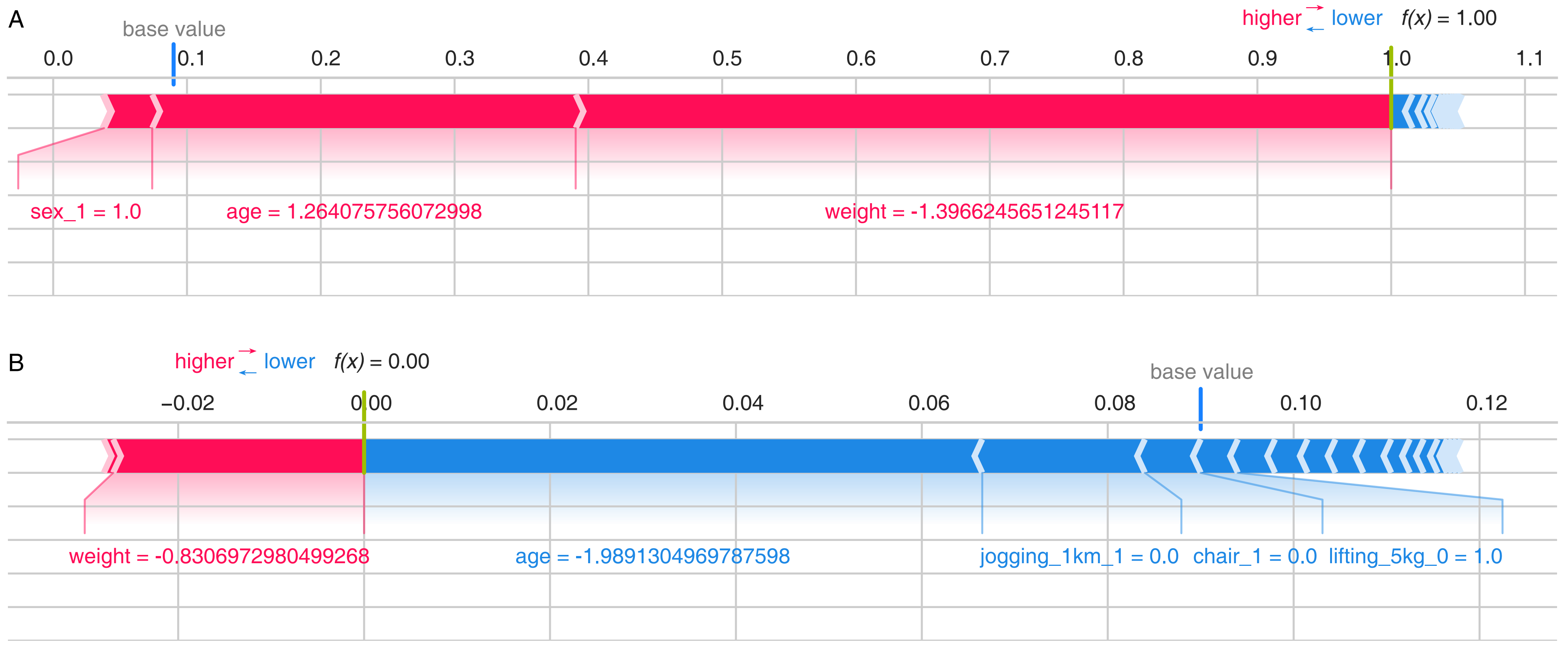

A
higher
lower
f(x) = 1.00
base value
0.0
0.1
0.2
0.3
0.4
0.5
0.6
0.7
0.8
0.9
1.0
1.1
sex_1 = 1.0
age = 1.264075756072998
weight = -1.3966245651245117
B
higher
lower
f(x) = 0.00
base value
−0.02
0.00
0.02
0.04
0.06
0.08
0.10
0.12
weight = -0.8306972980499268
age = -1.9891304969787598
jogging_1km_1 = 0.0
chair_1 = 0.0
lifting_5kg_0 = 1.0

Figure S6

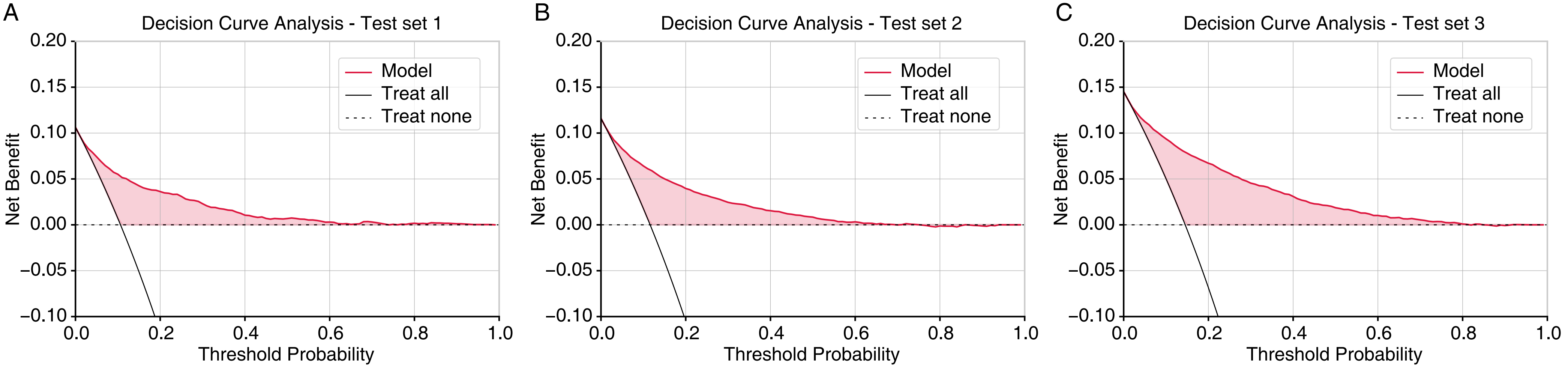
A
Decision Curve Analysis - Test set 1
B
Decision Curve Analysis - Test set 2
C
Decision Curve Analysis - Test set 3
Model
Treat all
Treat none
Net Benefit
Threshold Probability
0.20
0.15
0.10
0.05
0.00
−0.05
−0.10
0.0
0.2
0.4
0.6
0.8
1.0

Figure S7

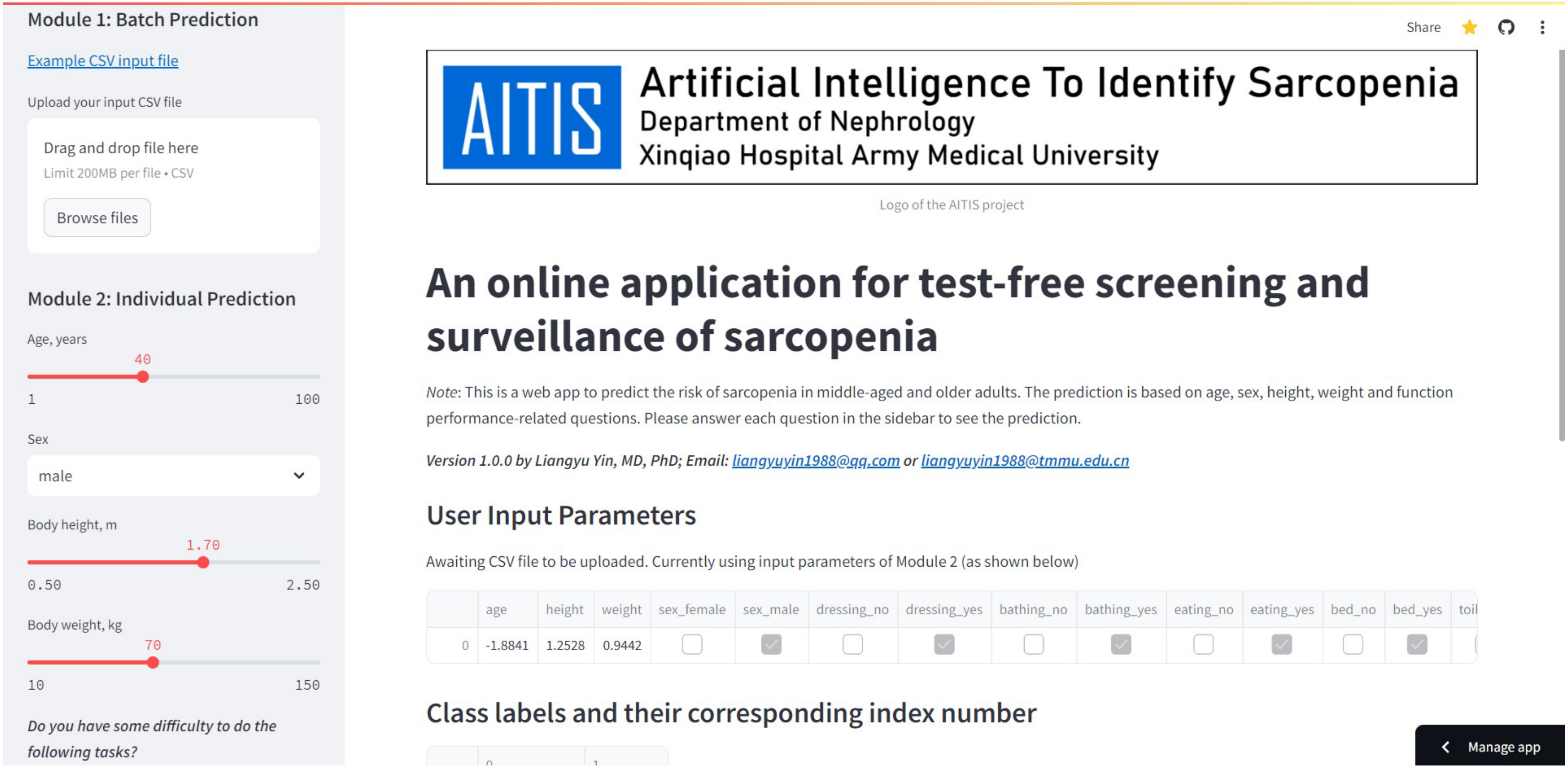

Module 1: Batch Prediction
Example CSV input file
Upload your input CSV file
Drag and drop file here
Limit 200MB per file • CSV
Browse files
Module 2: Individual Prediction
Age, years
40
1
100
Sex
male
Body height, m
1.70
0.50
2.50
Body weight, kg
70
10
150
Do you have some difficulty to do the following tasks?
Share
Artificial Intelligence To Identify Sarcopenia
Department of Nephrology
Xinqiao Hospital Army Medical University
AITIS
Logo of the AITIS project
An online application for test-free screening and surveillance of sarcopenia
Note: This is a web app to predict the risk of sarcopenia in middle-aged and older adults. The prediction is based on age, sex, height, weight and function performance-related questions. Please answer each question in the sidebar to see the prediction.
Version 1.0.0 by Liangyu Yin, MD, PhD; Email: liangyuyin1988@qq.com or liangyuyin1988@tmmu.edu.cn
User Input Parameters
Awaiting CSV file to be uploaded. Currently using input parameters of Module 2 (as shown below)
age height weight sex_female sex_male dressing_no dressing_yes bathing_no bathing_yes eating_no eating_yes bed_no bed_yes toil
0 -1.8841 1.2528 0.9442
Class labels and their corresponding index number
Manage app
